# Reviewed article: Campos-Rosa J, Puchivailo RAC, Schäfer AA, Meller FO, Miranda VIA. Ferrous Sulfate and Folic Acid Supplementation in Pregnant People in Primary Health Care: a Cross-Sectional Study, Criciúma, 2022. Epidemiol Serv Saude. 2026:35;e20240377

**DOI:** 10.1590/S2237-96222026v35e20240377.b

**Published:** 2025-11-28

**Authors:** Silvio Barberato-Filho

**Affiliations:** 1 Universidade de Sorocaba, Sorocaba, SP, Brazil Universidade de Sorocaba Sorocaba SP Brazil

## First round comments

Prezados autores,

Recebemos o parecer dos revisores e encaminhamos para análise e providências. Enfatizamos a necessidade de submeter a versão do manuscrito em word, para que possa ser avaliada a similaridade pelo iThenticate.

Além das indicações dos revisores, acrescentamos as seguintes observações/recomendações:

1. Na introdução, incluir citação e referência que fundamente a afirmação:
“No entanto, mesmo com um programa de abrangência universal, estudos apontam que a suplementação tanto com ferro quanto com ácido fólico está abaixo do recomendado, sendo 40 mg e 0,4 mg diariamente, respectivamente.”
2. Evite uso de “respectivo” ou “respectivamente”, seja na comparação com a literatura ou na apresentação de resultados - trazer os dados para próximo da sua correspondência, o que torna o texto mais claro para os leitores.
3. No método, substituir a citação que aparece como “(cidades.ibge.gov.br)” pelo respectivo número. Incluir na lista de referências usando o número sequencial (18).
4. Rever concordância verbal nos critérios de exclusão (p.4, linhas 47-51).
5. Em coleta de dados: especificar qual a Universidade. Rever redação: o que foi padronizado? No segundo parágrafo, mencionar que se trata de sistema informatizado. O uso do termo sistema de saúde pode apresentar ambiguidade.
6. Nas variáveis investigadas, corrigir: >=30. Não deixar espaços antes e depois de /. Rever no manuscrito todo. Incluir as variáveis de hábitos de vida.
7. Na discussão, corrigir redação em: “Por outro lado, um estudo de coorte no Maranhão com 5.110 puérperas apresentou prevalência de suplementação de sulfato ferroso em gestantes menores de 20 anos foi maior (16).”
8. Ainda na discussão, está correto associar o consumo de bebida alcoólica com a vulnerabilidade social? Isso se aplica ao estudo em questão?
9. Na afirmação “Dentre os pontos positivos, aponta-se a representatividade das gestantes atendidas na Atenção Primária a Saúde e...” a que se refere o termo representatividade?
10. Referências: ajustar rigorosamente às normas da revista e usar a forma abreviada na página final.
11. Não incluir espaço antes e após sinais (=, , ≤, ≥ etc.). Atenção às Tabelas.
12. Evitar o uso indiscriminado/desnecessário de artigos indefinidos (um, uma) ao longo do texto
13. Na Tabela 3, manter duas casas decimais no p-valor (três valores estão com uma casa decimal)
14. Usar crase, quando necessário.

Recommendation: Minor revision 

## Second round comments

Prezados autores,

Verificamos o empenho para atender às recomendações anteriores dos revisores. A maior parte foi corrigida, mas alguns pontos ainda requerem atenção:

1. Título: não ficou claro o motivo da indicação do ano 2023, uma vez que no método o período de coleta
aponta abril a dezembro de 2022 e as tabelas também mencionam o ano de 2022.
2. Medidas de dispersão: devem ter intervalos separados por ponto e vírgula e com espaço. Essa instrução
foi alterada após a revisão anterior. Exemplo: (IC95% 1,14; 2,23).
3. Na conclusão do resumo, sugere-se alterar a segunda frase para: A identificação dos fatores associados
pode orientar estratégias de intervenção mais direcionadas e eficazes por parte da gestão de saúde.
4. No método, conforme as recomendações do STROBE, indicar os esforços para abordar potenciais fonts de viés.
5. No método, grafar corretamente o nome do aplicativo RedCap - Research Electronic Data Capture.
6. Nas variáveis investigadas, houve um erro de interpretação no parecer anterior. A solicitação de correção referia-se ao sinal de menor (<) incluído equivocadamente no lugar do sinal de maior (>). Feita essa correção, manter o formato anterior do símbolo de maior ou igual convencional (≥) em todo o texto e tabelas.
7. Também no corpo do texto, grafar raça/cor da pele.
8. Evitar o uso indiscriminado de artigos indefinidos, como por exemplo em:
“... no Maranhão com 5.110 puérperas revelou uma maior prevalência...”
“... apresentou um aumento progressivo na suplementação de sulfato...”
“... encontraram um maior uso de sulfato ferroso entre gestantes mais jovens...”
9. Na Tabela 3, manter duas casas decimais no intervalo de confiança (três valores estão com uma casa
decimal). No parecer anterior havíamos mencionado que o erro estava no p-valor. Ainda na Tabela 3, excluir a letra a sobrescrita, uma vez que a nota de rodapé foi suprimida.
10. Certificar-se que as tabelas estejam dispostas corretamente. Nessa versão as Tabelas 1 e 2 aparecem
na mesma página no arquivo em pdf.
11. Nas Tabelas, alinhar os dados numéricos à direita e os de texto à esquerda.
12. Corrigir o número de todas as citações após a inserção da referência 18 (IBGE). Há muitos erros (exemplo: na discussão, nas citações 18, 19, 20, 24, 26 e provavelmente outras). Não consta citação da referência 25.
13. As referências estão, em sua maioria, fora das normas preconizadas pela RESS: não apresentam ano,
volume, número, intervalo de páginas, por exemplo. Corrigir rigorosamente.
14. Corrigir pontuação nos valores de renda mensal, no texto e nas Tabelas.
15. Na folha de rosto, as “afiliações dos autores” parecem continuar fora das normas da revista. Ver modelona instrução aos autores e em publicações recentes (Instituição, Unidade, Departamento ou Programa, Cidade, UF, País).
16. Corrigir o nome da autora Vanessa Iribarrem Avena Miranda, na folha de rosto.

Enfatizamos a necessidade de atender cuidadosamente às recomendações, em especial, quanto às citações, referências e formatação de tabelas.

Recommendation: Major revision

## Third round comments

Prezados autores,

Algumas questões apontadas anteriormente ainda não foram corrigidas de forma adequada. Favor verificar:

1. Tabelas - Ver instruções aos autores: “Títulos e notas de rodapé devem ficar fora das linhas de grade das tabelas”. Portanto, o título de cada tabela NÃO DEVE estar no corpo da Tabela (na primeira linha), mas acima dela.
2. Rever a numeração das citações a partir do número 18. No método (Contexto), a citação 18 refere-se à referência do IBGE. Na discussão, a citação 18 refere-se ao estudo transversal do município do Rio Grande (RS) - Referência 19. Revisar e corrigir todas as citações (e referências) a partir do número 18 (19, 20, 21, 22, 23, 24, 25, 26).
3. Rever redação (concordância, pontuação etc.) em:
a. “Aproximadamente metade delas tinham de 9 a 11 anos de estudo (50,5%). Cerca da metade tinha renda de R$1.001,00 a R$2.000,00 (45,0%).”
b. “A mesma tendência foi observada em relação a suplementação de ácido fólico, as gestantes que realizaram seis ou mais consultas de pré-natal apresentaram maior frequência de uso, atingindo 95,6%, enquanto aquelas que fizeram menos de seis consultas registraram 86,2% (p-valor 0,001).”
c. “Como é visto no estudo transversal realizado no município de Rio Grande, no Rio Grande do Sul, com 2.685 puérperas, demonstrou que 54,2% das puérperas realizaram a suplementação de ácido fólico, sendo que 85,8% realizaram seis ou mais consultas de pré-natal (15).”
4. Várias referências estão fora das normas preconizadas pela RESS: não apresentam número da revista, intervalo de páginas ou identificação do artigo eletrônico, por exemplo. Uniformizar formato da data de acesso. Checar identificação eletrônica do artigo da referência 4 (f3443-3 ou f3443?). Corrigir rigorosamente todas as referências.

Recommendation: Minor revision 

